# Expression, purification and characterization of a recombinant fusion protein based on the human papillomavirus-16 E7 antigen

**DOI:** 10.1186/2193-1801-2-12

**Published:** 2013-01-12

**Authors:** Milaid Granadillo, Aileen Batte, Victoria M Lugo, Alexis Musacchio, Mónica Bequet-Romero, Lázaro Betancourt, Vladimir Besada, Luis Javier, Raychel Molina, Viviana Falcón, Isis Torrens

**Affiliations:** Center for Genetic Engineering and Biotechnology, P.O. Box 6162, Cubanacan, Playa, Havana, 10600 Cuba

**Keywords:** Fusion protein, Human papillomavirus-16, E7 antigen, LALF_32-51_, *E. coli*

## Abstract

A fusion protein comprising a cell penetrating and immunostimulatory peptide corresponding to residues 32 to 51 of the *Limulus polyphemus* protein linked to human papillomavirus (HPV)-16 E7 antigen (LALF_32-51_-E7) was expressed in *E. coli* BL21 (DE3) cells. The recombinant protein in *E. coli* accounted for approximately 18% of the total cellular protein and purified with a single affinity chromatographic step. Yields of approximately 38 mg purified LALF_32-51_-E7 per liter of induced culture was obtained with an overall 52% recovery and constitutes a promising setting for the future production and scaling-up. Purified protein was characterized as soluble aggregates with molecular weight larger than 670 kDa, which is considered an important property to increase the immunogenicity of an antigen preparation. The recombinant fusion protein LALF_32-51_-E7 will be a promising vaccine candidate for the treatment of HPV-16 related malignancies.

## Background

Cervical cancer represents the second most frequent cancer in women (zur Hausen 2009). Today it is very well established that so-called high-risk human papillomavirus (HPV) infections, particularly those related to HPV-16, cause cervical cancer (zur Hausen 2002). The availability of preventive vaccines against HPVs represents a milestone in the prevention of this infection (Harper et al. 2006), but no effective therapeutic vaccine or immunological treatment exists for individuals already infected or for the 470,000 women that develop high-grade dysplasia, carcinoma *in situ*, and cervical cancer each year.

The oncogenic potential of HPV-16 is mainly ascribed to the viral oncoprotein E7, which has been shown to interact with a variety of cellular proteins (Munger et al. 2001). Moreover, being expressed in all the cervical tumors and in precancerous lesions, the E7 protein represents a specific target for immunotherapy (zur Hausen 2002).

We designed a fusion protein comprising a cell penetrating and immunostimulatory peptide corresponding to residues 32 to 51 of the *Limulus polyphemus* protein (LALF_32-51_) linked to HPV-16 E7 antigen (LALF_32-51_-E7) and selected *E. coli* as protein expression systems by its relative simplicity, its inexpensive and fast high-density cultivation, the well known genetics and the large number of compatible tools available for biotechnology (Jana and Deb 2005).

In a previous paper we describes some results related the biological properties of this fusion protein, a promissory vaccine candidate for the treatment of HPV-16-related malignancies (Granadillo et al. 2011). Here we describe the expression and purification and some results concerning the characterization of this recombinant fusion protein. We demonstrated that LALF_32-51_-E7 is highly expressed in *E. coli* BL21 (DE3) and easily purified with a single chromatographic step with a high purity. Non-optimized yields obtained by us are in order of 38 mg/l of bacterial culture, a very promising setting for the future production and scaling-up. We also show that the protein is obtained in a highly aggregated form, a property that is considered very important to increase the immunogenicity of an antigen preparation.

## Results

### Bacterial expression and purification of LALF_32-51_-E7 fusion protein

**Figure 1:**
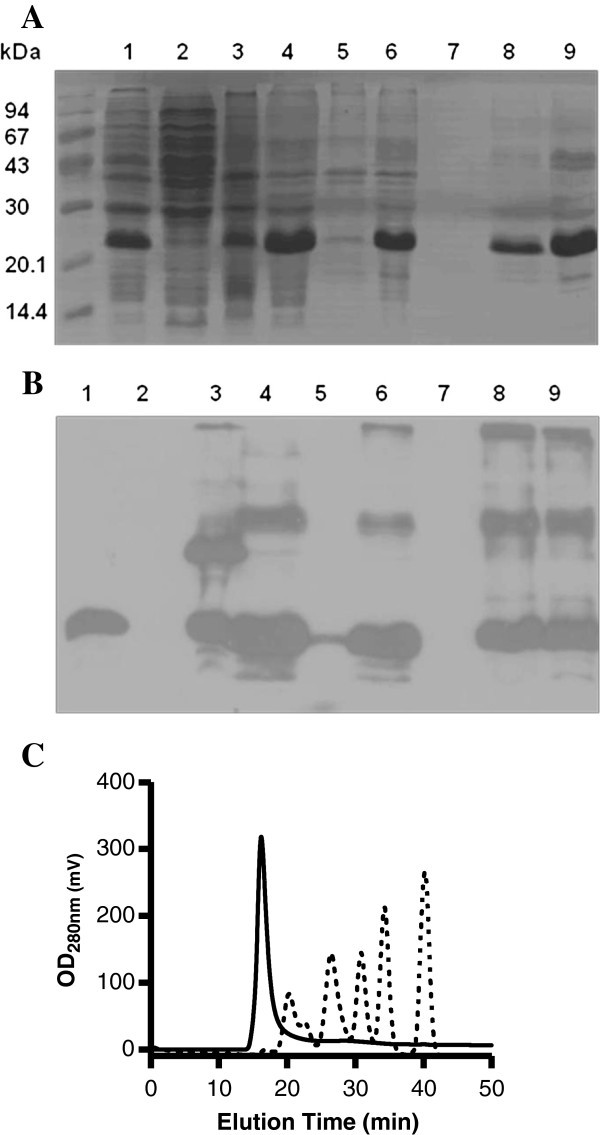
**Expression and purification of the fusion protein LALF**_**32-51**_**-E7. (A)** SDS-PAGE (15%). **(B)** Western Blot using anti-HPV-16 E7 monoclonal antibody: initial crude extract of *E. coli* proteins containing the expressed (about 18%) fusion protein LALF_32-51_-E7 (lane 1); soluble fraction after cell disruption (lane 2); insoluble fraction after cell disruption (lane 3); soluble fraction after treatment with 6 M urea (lane 4); insoluble fraction after treatment with 6 M urea (lane 5); IMAC purification, initial sample (lane 6); IMAC purification, wash with 10 mM imidazole (lane 7); IMAC purification, elution with 300 mM imidazole (lane 8); 300 mM imidazole elution fraction after desalting (lane 9). Molecular mass markers are indicated on the left. **(C)** Analytical gel filtration chromatogram of purified LALF_32-51_-E7 (continuous line). Dash line is representative of the retention time for (from left to right) bovine thyroglobulin (670 kDa), bovine gamma globulin (158 kDa), ovalbumin (44 kDa), myoglobin (17 kDa) and vitamin B-12 (1.35 kDa).

The DNA sequence of kanamycin resistance gene (KanR) was amplified by PCR from the corresponding gene of a reliable plasmid template, purified and cloned into pPEPE7M-7 vector (Granadillo et al. 2011), which expresses the 134 amino acid LALF_32-51_-E7 fusion protein. After corroborating that the KanR gene was successfully cloned, BL21 (DE3) cells were transformed with the pPEPE7M-7K plasmid and induced for expression obtaining approximately 7 g/l of biomass at the end of the fermentation process. As shown in Figure 1A, lane 1, LALF_32-51_-E7 accounted for approximately 18% of the total cellular protein and migrated as an approximately 24 kDa protein in 15% sodium dodecyl sulfate polyacrilamide electrophoresis (SDS-PAGE). The fusion protein was located in the insoluble fraction after cell disruption (Figure 1A, lane 3). This protein was solubilized from bacterial pellet using 6 M urea (Figure 1A, lane 4) and further purified by immobilized metal-ion affinity chromatography (IMAC) up to 94% purity (Figure 1A, lane 9). The fusion protein was recognized by an anti-HPV-16 E7 mouse monoclonal antibody in Western blot (Figure 1B). The 300 mM imidazole eluate contains a major 24 kDa LALF_32-51_-E7 band and high molecular weight (MW) aggregates of this same protein, as shown by Western Blot analysis (Figure 1B, lanes 8 and 9). Yields of approximately 38 mg purified LALF_32-51_-E7 per liter of induced culture was obtained with an overall 52% recovery (Table 1). The IMAC-purified fusion protein was further analyzed by size exclusion analytic HPLC in Superdex 200 10/300 GL. A major peak eluting in the void volume of the column and accounting for 100% of the applied protein was obtained (Figure 1C). According to the used column calibration standard, this peak appears to contain soluble aggregates with MW larger than 670 kDa.

**Table 1:** **Summary of LALF**_**32-51**_**-E7 purification (from dry weight of the biomass (0.75 g/l))**

Step	Volume (mL)	Total protein concentration (mg/mL)	Purity (%)^a^	Amount of LALF_32-51_-E7 (mg)	Purification fold	Step yield (%)	Overall yield (%)
Initial crude extract	30	5.85	18	31.59	1	100	100
Insoluble fraction after cell disruption	30	2.33	32	22.37	1.7	71	71
Solubilization	15	3.07	45	20.72	1.4	93	66
IMAC elution	25	0.75	94	17.63	2	85	56
Desalting	35	0.5	94	16.45	1	93	52

^a^ Determined by densitometry.

The protein concentration was determined by Bio-Rad protein assay (Bradford 1976), using bovine serum albumin as the reference standard.

### Transmission electron microscopy studies

**Figure 2:**
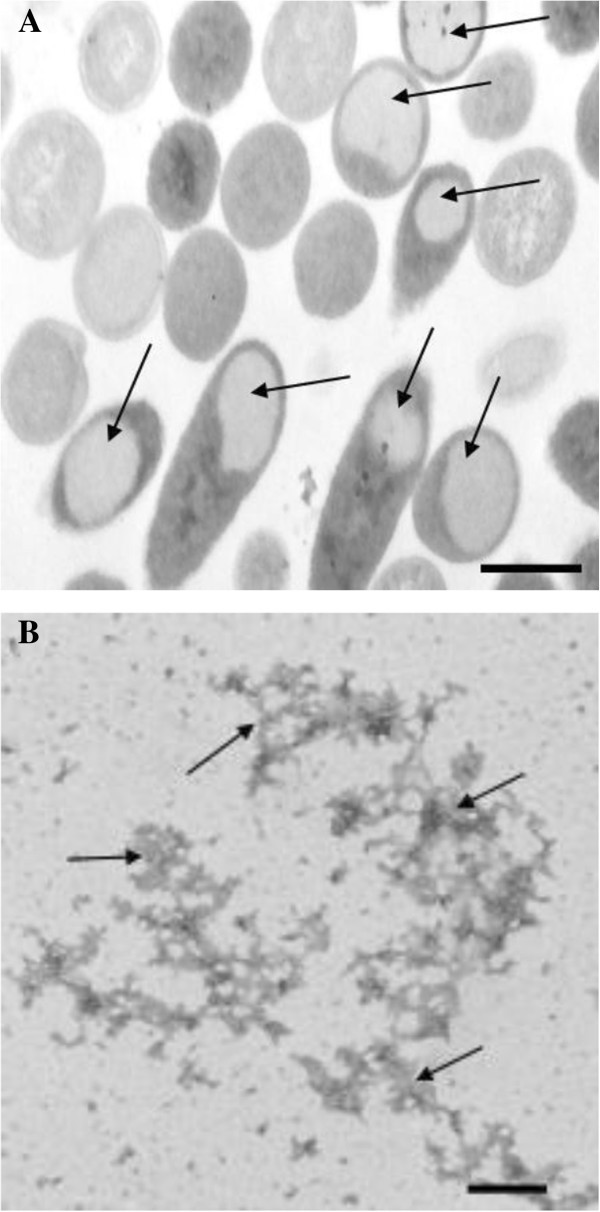
**Transmission electron micrographs.(A)** Microphotograph showing at ultrastructural level the presence of inclusion bodies inside the *E. coli* transformed cell, corresponding to the LALF_32-51_-E7 fusion protein. The bar represents 500 nm. **(B)** Electron micrograph of the negatively stained LALF_32-51_-E7 preparation sample showing aggregates of different shape and size. The bar represents 200 nm.

In order to corroborate if the LALF_32-51_-E7 fusion protein was expressed as inclusion bodies, ultrastructural studies were performed. As expected, transmission electron microscopy study of cells harboring pPEPE7M-7K indicated that the fusion protein is produced as cytoplasmic inclusion bodies (Figure 2A).

To characterize the purified fusion protein, preparations of LALF_32-51_-E7 were analyzed by negative staining**.** The Figure 2B shows representative electron microscopy micrograph of the LALF_32-51_-E7 preparation. The protein appears as aggregates of different shape and size.

### Mass spectrometry analysis

**Figure 3:**
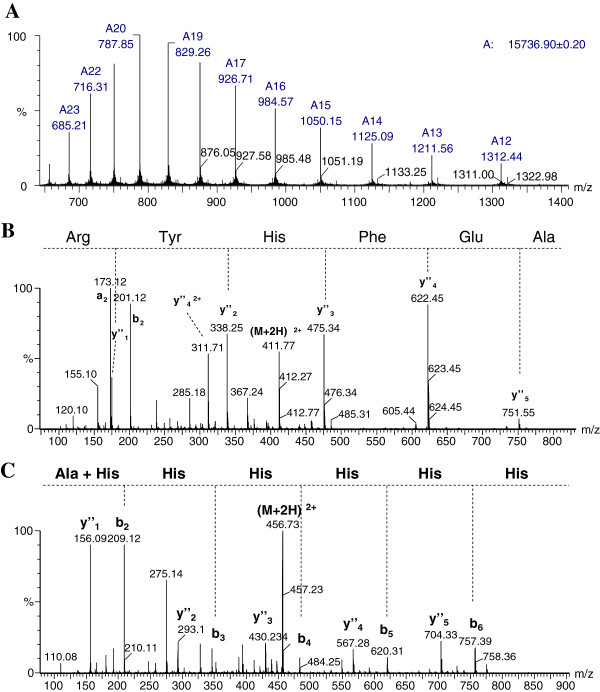
**Mass spectrometry analysis. (A)** ESI mass spectra of rcm-LALF_32-51_-E7. **(B)** Sequencing by ESI-MS/MS of the peptide of m/z 411.70 corresponding to the N-terminal of LALF_32-51_-E7. **(C)** Sequencing by ESI-MS/MS of the peptide of m/z 456.70 corresponding to the C-terminal of LALF_32-51_-E7.

**Figure 4:**
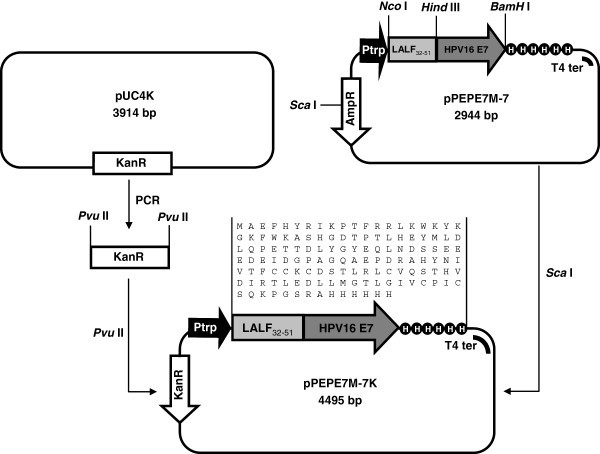
**Schematic representation of pPEPE7M-7K construction for the expression of the LALF**_**32-51**_**-E7 fusion protein.**

According to the gene sequence, LALF_32-51_-E7 is synthesized as a protein of 134 amino acids containing a hexa-histidine tag at the C-terminus, with a theoretical mass value of 15867.85 Da. ESI-MS of reduced and carboamidomethylated protein (rcm- LALF_32-51_-E7) gave a major signal of 15736.90 Da in mass (Figure 3A), which is in good agreement with the theoretical value (15736.65 Da) calculated for the sequence starting from the second amino acid (alanine, abbreviated A in Figure 4). The major signal obtained differs in 130.95 mass units with respect to the expected theoretical mass of entire protein (15867.85 Da), indicating a full processing of the initiation methionine from the protein.

To further verify the identity of the molecule, the protein was enzymatically digested with trypsin, and generated fragments were analyzed by mass spectrometry. Identified peptides accounted for 93% of the entire sequence of LALF_32-51_-E7 (Table 2). Undetected peptides corresponded to fragments with less than 3 amino acids which are out of the mass range analysis of the mass spectrometer (400–2000 Th). ESI-MS/MS sequencing of N- and C-terminal suspected peptides confirm the lack of the initiation methionine (Figure 3B) and the presence of the six-His-tag, respectively (Figure 3C).

**Table 2:** **Observed m/z**^**a**^**values of the tryptic peptides derived from rcm-LALF**_**32-51**_**-E7 and their theoretical values calculated from the deduced sequence**

Observed m/z	Theoretical m/z	Charge	Sequence
411.70	411.70	2	2-7^b^
761.44	761.47	1	8-13
480.25	480.26	1	23-25
1396.20	1396.20	4	26-75
706.83	706.83	2	76-86
751.35	751.34	1	87-92
664.34	664.34	2	93-103
882.46	882.45	3	104-127
1124.60	1124.57	2	103-123
416.22	416.23	1	124-127
456.70	456.71	2	128-134^c^

^a^ Mass to charge ratio.

^b^ N-terminal peptide.

^c^ C-terminal peptide.

## Discussion

In this paper, we describe the expression, purification and some results related to the characterization of LALF_32-51_-E7 fusion protein; a promising vaccine candidate for the treatment of HPV-16 related malignancies. Antigen design was based on mutated version of viral HPV-16 E7 antigen bearing a base substitution of T by G in the triplet encoding cysteine at position 24 (substitution of Cys to Gly) in order to disrupt their binding to protein Rb (Munger et al. 1989; Barbosa et al. 1990; Jones et al. 1990), in this way reducing possible regulatory objections in the future development of a human vaccine candidate. To improve safety, and since the ampicillin resistance gene (AmpR) is precluded for use in humans, we introduced the KanR gene as a selectable marker of our final expression vector pPEPE7M-7K. The KanR gene is the antibiotic resistance marker often used while the AmpR is not acceptable due to concerns with hyper reactivity of some patients to β lactam antibiotics (Williams et al. 2009).

It is well documented that a key aspect influencing on the expression of heterologous proteins in *E. coli* cytoplasm is the selection of host strain (Sorensen and Mortensen 2005). In this sense *E. coli* BL21 (DE3) is the most common host and has proven outstanding in standard recombinant expression application, is able to grow vigorously in minimal media but however non-pathogenic and unlikely to survive in host tissues and cause disease (Chart et al. 2000). In this paper, we show that the fusion protein LALF_32-51_-E7 is highly expressed (18%) in *E. coli* BL21 (DE3). In agreement with our results other researchers have expressed efficiently recombinant fusion proteins for therapeutic purposes based on HPV-16 E7 antigen in *E. coli* (Chu et al. 2000; Preville et al. 2005; Liu et al. 2008). In this work we also show that LALF_32-51_-E7 was easily purified with a single affinity chromatographic step (up to 94% purity) that can be followed by other polishing ones (i.e. gel filtration) if manufactured for human vaccine purposes. The non-optimized yields obtained by us are in order of 38 mg of purified LALF_32-51_-E7 per liter of induced culture, a promising figure in terms of production and scaling-up. This study also reports that the fusion protein was obtained highly aggregated, a property that could be convenient to enhance immunogenicity for an antigen preparation. Other researchers have reported that while low MW aggregates such as dimers and trimers appear inefficient in inducing immune responses, large multimers whose MW exceeds 100 kDa are efficient inducers of immune responses (Rosenberg 2006). As our aim was to obtain a highly immunogenic E7 preparation, we did not focus on obtaining aggregates of identical shape and size considering that particles of different size can be taken up by different types of antigen presenting cells, such as dendritic cells, macrophages and polymorphonuclear leukocytes, sustaining a more potent immune response (Oyewumi et al. 2010).

In this study we also characterized the LALF_32-51_-E7 fusion protein by mass spectrometry. LALF_32-51_-E7 is a 134 amino acids protein with a hexa-histidine tag at the C-terminus and a theoretical molecular mass of 15867.85 Da. The mass spectrometry analyses were in good agreement with the theoretical molecular mass value of the full length gene product without the N-terminal methionine and verified the identity of the molecule.

Although the theoretical mass value of LALF_32-51_-E7 is approximately 16 kDa, the protein migrated in SDS-PAGE under reducing conditions as an approximately 24 kDa protein that is larger in size than predicted. This abnormal migration pattern has been previously reported for the HPV-16 E7 protein, and is attributable to their high content of acidic amino acid residues (Armstrong and Roman 1993; Bolhassani et al. 2008).

The *E. coli* expressed proteins represent a well-studied and cost-effective means for the production of vaccines. Our vaccine candidate represents not only a good substrate for antigen-presenting cell uptake and processing, but also a cost-effective promising approach for developing a HPV therapeutic vaccine. A generation of new low-cost HPV vaccines could represent the only possibility for women living in developing countries to gain access to HPV vaccination programs to prevent or treat pre-cancerous lesions and cancer.

## Conclusions

This is a report of a non-optimized process about the expression, purification and characterization of a recombinant fusion protein that is a cost-effective promising approach for developing a HPV therapeutic vaccine.

## Methods

### Fusion protein expression vector

The AmpR gene of the plasmid pPEPE7M-7 (Granadillo et al. 2011) that containing the recombinant LALF_32-51_-E7 fusion protein was interrupted by digestion with ScaI and a DNA fragment containing the KanR gene was cloned in this plasmid. The KanR gene was PCR amplified from pUC4K using two primers. The forward primer 5’ CAG CTG GCC ACG TTG TGT CTC AAA ATC 3’ contains a PvuII site as well as the reverse primer 5’ CAG CTG TTC AAC AAA GCC GCC GTC CC 3’. The PCR product was digested with PvuII, purified and ligated to pPEPE7M-7 which had been cut with ScaI. The KanR gene orientation was verified by ClaI digestion. Clones resulting in bands of approximately 3.2 and 1.3 kb have opposite orientation respect to the target gene and clones resulting in bands of 2.5 and 2 kb have the same orientation respect to the target gene. We selected the clone in which the KanR gene has opposite orientation respect to the target gene and was designated pPEPE7M-7K (Figure 4).

### Expression and purification of LALF_32-51_-E7

BL21 (DE3) cells transformed with pPEPE7M-7K were inoculated in 500 ml of LB medium containing kanamycin (50 μg/ml) and incubated for 6 h at 37°C in a shaker. This culture were used to inoculate a 5 l fermentor (B.E. Marubishi, Japan) containing M9 salt medium enriched with 10 g/l casein hydrolysate, 0.011 g/l CaCl_2_ · 2H_2_O, 0.246 g/l MgSO_4_ · 7H_2_O, 2 g/l glucose and 0.05 g/l kanamycin. After three h, 3 β-indole-acrylic acid was added to a final concentration of 0.04 g/l and the culture was grown for another 15 h to obtain as much possible recombinant protein as insoluble inclusion bodies according to the standard production protocol for *E coli* based technology implemented in our institute. The fermentation parameters were pH 7.0, 37°C, 350 rpm and 1.0 vvm aeration rate, operated in a batch mode.

Cells were harvested by centrifugation at 15 000 *g* for 20 min at 4°C. Then, 3 g of cellular biomass was resuspended in 30 ml of rupture buffer (50 mM NaH_2_PO_4_, 300 mM NaCl, pH 8.0) at a ratio of biomass/buffer of 1:10. The biomass was disrupted in French Press (Othake, Japan) at 1500 bar, with two passes on 4°C. After centrifugation at 15 000 *g* for 30 min at 4°C, the pellet was recovered and the recombinant protein was totally solubilized in 6 M urea in carbonate-bicarbonate buffer pH 10.6. Cell debris was removed by centrifugation at 15 000 *g* for 30 min at 4°C and the soluble fraction containing the fusion protein, that have a six-histidine C-terminus tail for purification purposes, was recovered. Due to the recombinant protein was totally solubilized in 6 M urea in carbonate-bicarbonate buffer pH 10.6 and not in other buffers at lowers pH and at different urea concentrations (data not shown), the purification of the protein was necessarily conducted in carbonate-bicarbonate buffer pH 10.6. The soluble fraction was diluted in equal volume of 1 M NaCl and loaded onto a 22 ml His-Select® Nickel Affinity Gel (Sigma, Catalog number P6611) equilibrated with loading buffer (3 M urea and 0.5 M NaCl in carbonate-bicarbonate pH 10.6). The column was then washed with loading buffer containing 10 mM imidazole and the protein of interest was eluted with 300 mM imidazole. The eluted IMAC fraction (25 ml) was further loading onto a HiPrep 26/10 desalting column (GE Healthcare) equilibrated with 10 mM Tris pH 8.0 renaturation buffer and following the manufacturer’s instructions. In this chromatographic step the protein was refolded because the urea and imidazole were totally removed. The peak fractions containing LALF_32-51_-E7 protein were pooled after desalting and then endotoxin removal was performed using an EndoClean™ Kit from BioVintage. The final protein preparation contained <0.05 endotoxin units (EU)/μg as measured by the chromogenic Limulus ameobocyte lysate assay (Associates of Cape Cod, Inc). Samples in each step of the process were collected and later analyzed by SDS-PAGE and Western blot. The MW of the recombinant protein was estimated using size exclusion analytic HPLC (YL9100) in a Superdex 200 10/300 GL column (GE Healthcare); briefly 200 μL of the purified protein were applied at a flow rate of 0.5 ml/min in 10 mM Tris (pH 8.0). MW was estimated using the retention times, in comparison with a gel filtration standard preparation (Bio-Rad).

### SDS-PAGE and Western blot

SDS-PAGE (15%) was performed according to Laemmli (Laemmli 1970). Protein expression and purity were evaluated by densitometry (TDI-1D manager 2.0 software, Spain) of SDS-PAGE gels. Proteins were visualized by Coomasie blue staining. The identity of the protein was verified by Western blot (Burnette 1981). Briefly, proteins were separated by SDS-PAGE and transferred to Hybond C extra nitrocellulose (Amersham Biosciences) in a semi-dry transfer unit (Bio-Rad). The membrane was blocked with 5% skimmed milk in PBS (140 mM NaCl, 2.7 mM KCl, 10 mM Na_2_HPO_4_ and 1.8 mM KH_2_PO_4_, pH 7.4)/0.1% Tween 20 (PBS-T) overnight at 4°C. After three washes with PBS-T, the membrane was incubated for 2 h at 25°C with mouse anti-HPV-16 E7 monoclonal antibody (Abcam) diluted 1:1000 in blocking solution. The membrane was washed three times with PBS-T and further incubated for 1 h at 25°C with goat anti-mouse IgG antibodies conjugated with HRP (Sigma) diluted 1:5000 in blocking solution. After extensively washing with PBS-T, the antibody-reactive bands were detected with an enhanced chemiluminescence kit (Amersham Pharmacia Biotech).

### Transmission electron microscopy

For ultrastructural studies the samples consisting of pelleted cells (2 x 10^7^ cells) from non-transformed or transformed BL21 (DE3) *E. coli* that express the recombinant fusion protein were fixed with 3.2% glutaraldehyde, and post-fixed for 1 h in 2% OsO_4_. Then it was rinsed with 0.1 M PBS, pH 7.2, and dehydrated in increasing ethanol concentrations such as 50%, 70%, 80%, 90% and 100%. The embedding was in Spurr. The blocks were sectioned with an ultramicrotome (NOVA, LKB), and the ultrathin sections were placed on 400-mesh cooper grids. The ultrathin sections were stained with uranyl acetate and lead citrate and then examined in a JEOL-JEM 2000 EX electron microscope (JEOL, Japan).

For the negative staining studies, a drop of purified LALF_32-51_-E7 fusion protein was placed on to a 400-mesh copper grid coated with formvar-carbon film. Following 30 min of sample absorption and washing with water, grids were stained for 30 s with 2% uranyl acetate. After staining, grids were dried with Whatman no. 1 filter paper and allowed to air dry for 15 min. Samples were then viewed on JEOL-JEM 2000 EX electron microscope.

### Reduction and S-carboamidomethylation of cysteines

The LALF_32-51_-E7 protein (10 nmoles) was dissolved in 100 ml of 6 M guanidium chloride, 500 mM Tris, pH 8.1, and incubated for 2 h with dithiothreitol (DTT) 50-fold in excess over cysteines, in a nitrogen atmosphere at 37°C. Iodoacetamide was added 2-fold over DTT, and the reaction proceeded at 25°C for 30 min in the dark. The reduced and fully alkylated protein (rcm-LALF_32-51_-E7) was desalted by HPLC system (LKB-Phamacia, Sweeden) in a RP-C4 column (4.6 x 50 mm, Vydac). The elution was performed with a linear gradient of solvent B (0.05% TFA in acetonitrile) from 5 to 60% in 30 min at a flow rate of 0.8 ml/min (Solvent A, 0.1% TFA in water). The eluate was monitored at 226 nm. An aliquot was submitted to ESI-MS analysis and the rest of the sample was evaporated under vacuum to dryness before trypsin digestion.

### Trypsin digestion

The rcm-LALF_32-51_-E7 was reconstituted in 0.05% NH_4_HCO_3_ buffer and digested with trypsin (Promega, USA) at enzyme-to substrate mass ratio 1:50 for 4 h at 37°C.

### Mass spectrometry

ESI-MS and MS/MS spectra were acquired using a hybrid quadrupole orthogonal acceleration tandem mass spectrometer QTOF-2™ from Micromass (Manchester, UK) fitted with a Z-spray nanoflow electrospray ion source. Other measuring conditions and data processing were the same as reported previously (Gonzalez et al. 2003).

## Abbreviations

HPV: Human papillomavirus
KanR: Kanamycin resistance gene
MW: Molecular weight
SDS-PAGE: Sodium dodecyl sulfate polyacrilamide electrophoresis.

## References

[CR1_104] Armstrong DJ Roman A The anomalous electrophoretic behavior of the human papillomavirus type 16 E7 protein is due to the high content of acidic amino acid residues Biochem Biophys Res Commun 1993 192 1380 1387 10.1006/bbrc.1993.1569 8389548

[CR2_104] Barbosa MS Edmonds C Fisher C Schiller JT Lowy DR Vousden KH The region of the hpv E7 oncoprotein homologous to adenovirus E1a and Sv40 large T antigen contains separate domains for R binding and casein kinase II phosphorilation EMBO J 1990 9 153 160 2153075 10.1002/j.1460-2075.1990.tb08091.x PMC551641

[CR3_104] Bolhassani A Zahedifard F Taghikhani M Rafati S Enhanced immunogenicity of HPV16E7 accompanied by Gp96 as an adjuvant in two vaccination strategies Vaccine 2008 26 3362 3370 10.1016/j.vaccine.2008.03.082 18471945

[CR4_104] Burnette WN "Western blotting": electrophoretic transfer of proteins from sodium dodecyl sulfate–polyacrylamide gels to unmodified nitrocellulose and radiographic detection with antibody and radioiodinated protein A Anal Biochem 1981 112 195 203 10.1016/0003-2697(81)90281-5 6266278

[CR5_104] Chart H Smith HR La Ragione RM Woodward MJ An investigation into the pathogenic properties of Escherichia coli strains BLR, BL21, DH5alpha and EQ1 J Appl Microbiol 2000 89 1048 1058 10.1046/j.1365-2672.2000.01211.x 11123478

[CR6_104] Chu NR Wu HB Wu T Boux LJ Siegel MI Mizzen LA Immunotherapy of a human papillomavirus (HPV) type 16 E7-expressing tumour by administration of fusion protein comprising Mycobacterium bovis bacille Calmette-Guerin (BCG) hsp65 and HPV16 E7 Clin Exp Immunol 2000 121 216 225 10.1046/j.1365-2249.2000.01293.x 10931134 PMC1905702

[CR7_104] Gonzalez LJ Castellanos-Serra L Badock V Diaz M Moro A Perea S Santos A Paz-Lago D Otto A Muller EC Kostka S Wittmann-Liebold B Padron G Identification of nuclear proteins of small cell lung cancer cell line H82: An improved procedure for the analysis of silver-stained proteins Electrophoresis 2003 24 237 252 10.1002/elps.200390020 12652596

[CR8_104] Granadillo M Vallespi MG Batte A Mendoza O Soria Y Lugo VM Torrens I A novel fusion protein-based vaccine comprising a cell penetrating and immunostimulatory peptide linked to human papillomavirus (HPV) type 16 E7 antigen generates potent immunologic and anti-tumor responses in mice Vaccine 2011 29 920 930 10.1016/j.vaccine.2010.11.083 21145912

[CR9_104] Harper DM Franco EL Wheeler CM Moscicki AB Romanowski B Roteli-Martins CM Jenkins D Schuind A Costa Clemens SA Dubin G Sustained efficacy up to 4.5 years of a bivalent L1 virus-like particle vaccine against human papillomavirus types 16 and 18: follow-up from a randomised control trial Lancet 2006 367 1247 1255 10.1016/S0140-6736(06)68439-0 16631880

[CR10_104] Jana S Deb JK Strategies for efficient production of heterologous proteins in Escherichia coli Appl Microbiol Biotechnol 2005 67 289 298 10.1007/s00253-004-1814-0 15635462

[CR11_104] Jones RE Wegrzyn RJ Patrick DR Balishin NL Vuocolo GA Riemen MW Defeo-Jones D Garsky VM Heimbrook DC Oliff A Identification of HPV-16 E7 peptides that are potent antagonists of E7 binding to the retinoblastoma suppressor protein J Biol Chem 1990 265 12782 12785 2198278

[CR12_104] Laemmli UK Cleavage of structural proteins during the asseembly of the head of bacteriophage T4 Nature 1970 227 680 685 10.1038/227680a0 5432063

[CR13_104] Liu B Ye D Song X Zhao X Yi L Song J Zhang Z Zhao Q A novel therapeutic fusion protein vaccine by two different families of heat shock proteins linked with HPV16 E7 generates potent antitumor immunity and antiangiogenesis Vaccine 2008 26 1387 1396 10.1016/j.vaccine.2007.12.034 18272260

[CR14_104] Munger K Werness BA Dyson N Phelps WC Harlow E Howley PM Complex formation of human papillomavirus E7 proteins with the retinoblastoma tumor supressor gene product EMBO J 1989 8 4099 4105 2556261 10.1002/j.1460-2075.1989.tb08594.x PMC401588

[CR15_104] Munger K Basile JR Duensing S Eichten A Gonzalez SL Grace M Zacny VL Biological activities and molecular targets of the human papillomavirus E7 oncoprotein Oncogene 2001 20 7888 7898 10.1038/sj.onc.1204860 11753671

[CR16_104] Oyewumi MO Kumar A Cui Z Nano-microparticles as immune adjuvants: correlating particle sizes and the resultant immune responses Expert Rev Vaccines 2010 9 1095 1107 10.1586/erv.10.89 20822351 PMC2963573

[CR17_104] Preville X Ladant D Timmerman B Leclerc C Eradication of established tumors by vaccination with recombinant Bordetella pertussis adenylate cyclase carrying the human papillomavirus 16 E7 oncoprotein Cancer Res 2005 65 641 649 15695409

[CR18_104] Rosenberg AS Effects of protein aggregates: an immunologic perspective AAPS J 2006 8 E501 E507 10.1208/aapsj080359 17025268 PMC2761057

[CR19_104] Sorensen HP Mortensen KK Advanced genetic strategies for recombinant protein expression in Escherichia coli J Biotechnol 2005 115 113 128 10.1016/j.jbiotec.2004.08.004 15607230

[CR20_104] Williams JA Carnes AE Hodgson CP Plasmid DNA vaccine vector design: impact on efficacy, safety and upstream production Biotechnol Adv 2009 27 353 370 10.1016/j.biotechadv.2009.02.003 19233255 PMC2693335

[CR21_104] zur Hausen H Papillomaviruses and cancer: from basic studies to clinical application Nat Rev Cancer 2002 2 342 350 10.1038/nrc798 12044010

[CR22_104] zur Hausen H Papillomaviruses in the causation of human cancers - a brief historical account Virology 2009 384 260 265 10.1016/j.virol.2008.11.046 19135222

